# Differential Contribution of Rod and Cone Circadian Clocks in Driving Retinal Melatonin Rhythms in *Xenopus*

**DOI:** 10.1371/journal.pone.0015599

**Published:** 2010-12-20

**Authors:** Naoto Hayasaka, Silvia I. LaRue, Carla B. Green

**Affiliations:** Department of Biology, University of Virginia, Charlottesville, Virginia, United States of America; Pennsylvania State University, United States of America

## Abstract

**Background:**

Although an endogenous circadian clock located in the retinal photoreceptor layer governs various physiological events including melatonin rhythms in *Xenopus laevis*, it remains unknown which of the photoreceptors, rod and/or cone, is responsible for the circadian regulation of melatonin release.

**Methodology/Principal Findings:**

We selectively disrupted circadian clock function in either the rod or cone photoreceptor cells by generating transgenic *Xenopus* tadpoles expressing a dominant-negative CLOCK (XCLΔQ) under the control of a rod or cone-specific promoter. Eyecup culture and continuous melatonin measurement revealed that circadian rhythms of melatonin release were abolished in a majority of the rod-specific XCLΔQ transgenic tadpoles, although the percentage of arrhythmia was lower than that of transgenic tadpole eyes expressing XCLΔQ in both rods and cones. In contrast, whereas a higher percentage of arrhythmia was observed in the eyes of the cone-specific XCLΔQ transgenic tadpoles compare to wild-type counterparts, the rate was significantly lower than in rod-specific transgenics. The levels of the transgene expression were comparable between these two different types of transgenics. In addition, the average overall melatonin levels were not changed in the arrhythmic eyes, suggesting that CLOCK does not affect absolute levels of melatonin, only its temporal expression pattern.

**Conclusions/Significance:**

These results suggest that although the Xenopus retina is made up of approximately equal numbers of rods and cones, the circadian clocks in the rod cells play a dominant role in driving circadian melatonin rhythmicity in the *Xenopus* retina, although some contribution of the clock in cone cells cannot be excluded.

## Introduction

Vertebrate circadian clocks are distributed in a wide variety of tissues, where they generate local rhythms in many critical pathways that are fundamental for the proper physiology of each tissue (e.g., [1]–[5]. Previous studies have shown that the vertebrate retina has an autonomous circadian clock that drives many parameters of retinal physiology such as melatonin and dopamine synthesis, outer segment disc shedding of the photoreceptors, retinomotor movement, and light sensitivity (reviewed in [6]–[10]. The circadian clock located in the retina is unique since, in addition to containing all the components necessary for a complete ‘circadian system’ (i.e. a light input pathway, circadian oscillator, and multiple output pathways), it also serves as a direct input that delivers light information to the master clock in the suprachiasmatic nucleus (SCN) in the brain. Furthermore, recent studies have revealed that the mammalian retinal clock influences the master circadian pacemaker in the SCN in ways beyond simple entrainment, since rhythmic properties of the SCN are altered in enucleated mice or in mice with a retina-specific genetic clock ablation [4], [10]–[13].

*Xenopus laevis* has been a useful animal model for studying retinal physiology, and the *Xenopus* retina has been well characterized in terms

The next important question in understanding the circadian system that exists within the retina is to determine where the clock is located within the photoreceptor layer. The *Xenopus* retina contains approximately equal numbers of rods and cones and these cells are electrically coupled [20]. In this study, we address this issue by generating transgenic *Xenopus* that lack functional clocks specifically in either rod or cone photoreceptor cells. Our findings suggest that although both these cell types contain clock gene expression, the clocks in the rod cells are predominantly responsible for driving melatonin rhythms in the retina.

## Results

### Rod- or cone-specific expression of the dominant-negative XCLΔQ

**Figure 1:**
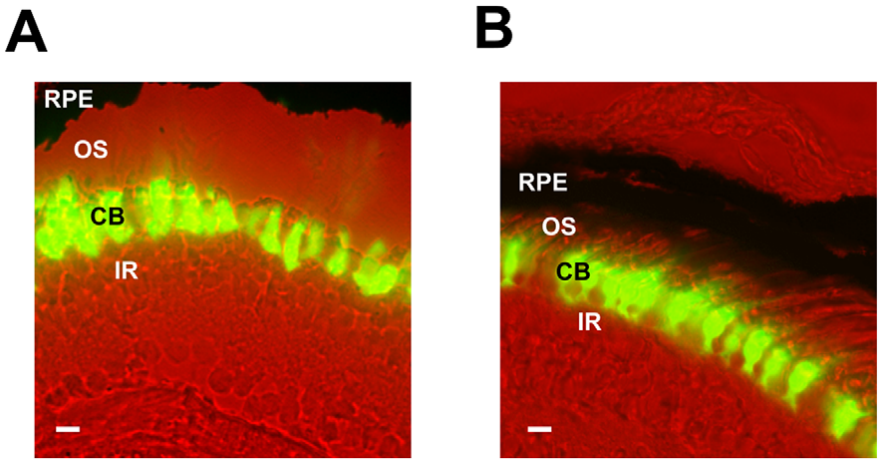
XCLΔQ-GFP expression in the specific cell-types in the transgenic photoreceptors. A. An image of a section from the XOP-XCLΔQ-GFP tadpole retina. XCLΔQ-GFP is observed only in the cell bodies and inner segments of the rod photoreceptor cells. B. In a CAR-XCLΔQ-GFP transgenic retina, GFP accumulates only in the cone photoreceptor cells. CB: photoreceptor cell body; OS: photoreceptor outer segment; IR: inner retina; RPE: retinal pigment epithelium. The scale bar indicates 10 µm.

We have previously reported that overexpression of a dominant negative *Xenopus* CLOCK (XCLΔQ; lacking the transactivation domain of normal CLOCK) in all retinal photoreceptors in *Xenopus* resulted in abolishment of the circadian melatonin rhythmicity [15]. To further investigate how each of the two retinal photoreceptor cell types in *Xenopus* contributes to the circadian rhythmicity, we generated groups of transgenic animals expressing XCLΔQ driven by one of two different promoters: the rod opsin promoter (XOP; [21], which drives rod-specific expression, and the cone arrestin promoter (CAR; [16], which drives cone-specific expression. Both transgenes were designed to express a XCLΔQ/EGFP fusion protein (named XOP-XCLΔQ-GFP and CAR-XCLΔQ-GFP, respectively), which has previously been shown to abolish core clock function both *in vitro* and *in vivo* [15]. After generating transgenic tadpoles using the modified REMI method [15], [22], we sectioned transgenic retinas and observed GFP fluorescence to verify that each transgene is expressed in the appropriate cell type in the photoreceptor layer. The XCLΔQ-GFP signal was detected only in the cell bodies and nuclei of the rod cells in the XOP-XCLΔQ-GFP retinas (Fig. 1A), and only in the cone cells in the CAR-XCLΔQ-GFP retinas (Fig. 1B). No GFP fluorescence above background was detected in any other cell types in the retina. As we have previously reported, the XCLΔQ-GFP expression did not alter the morphology of the photoreceptor cells at the light microscopy level. Also, as we observed in the previous transgenic study, a range of levels of GFP signal was observed among the individual transgenic animals, ranging from animals with high expression to some with undetectable expression [15].

### Rod-specific expression of the XCLΔQ alters melatonin rhythms in the retina

**Figure 2:**
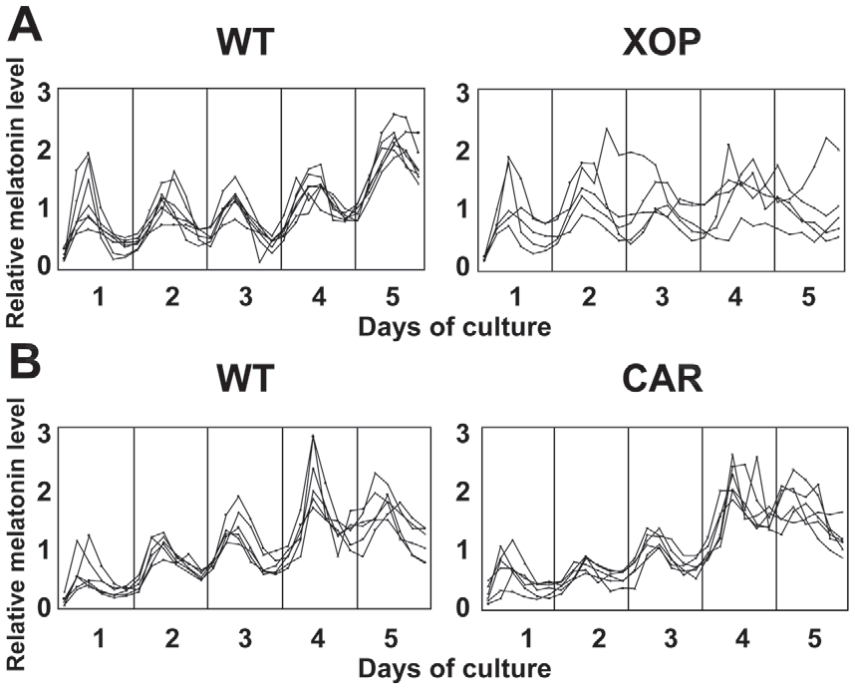
Melatonin release from the XOP-XCLΔQ-GFP and the CAR-XCLΔQ-GFP transgenic eyecups and wild-type controls. Each pair of eyecups was prepared from individual tadpoles and flow-through culture was performed for 5 days. Media fractions were collected every four hours, and assayed for melatonin by RIA. Each line represents melatonin release from a pair of eyecups. A. Melatonin release rhythms in the individual XOP-XCLΔQ-GFP eyecups (n = 5) and wild-type controls (n = 7). As compared to the wild-type eyes that demonstrate melatonin release in a circadian manner for five days, the majority of the transgenic eyes do not show significant circadian melatonin rhythmicity. B. Melatonin rhythms in the CAR-XCLΔQ-GFP (n = 6) and wild-type eyecups (n = 6). With some exceptions, eyecups release melatonin in a circadian manner.

**Figure 3:**
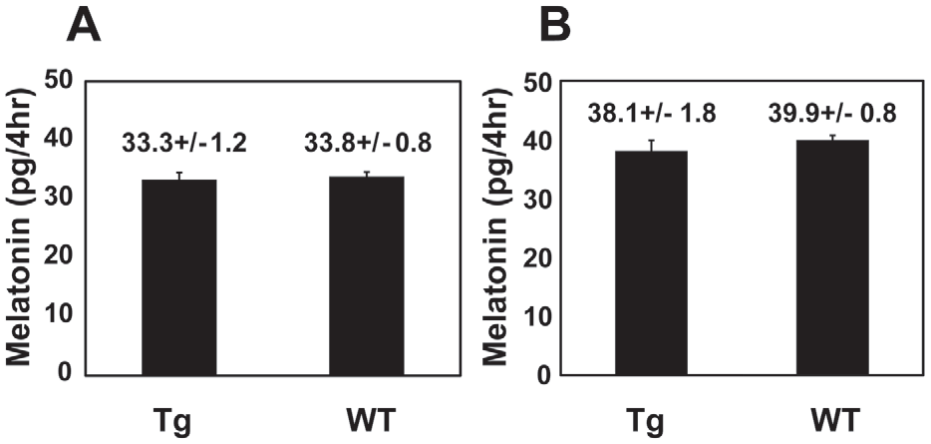
Total melatonin levels of the transgenic eyes and wild-type controls were comparable. Average of all fractions from the transgenic and wild-type eyes was calculated for the two different genotypes (XOP and CAR). Values on the figure are average melatonin content (picograms per 4hr) +/− SEM. A. The XOP transgenic (n = 25) vs. wild-type eyes (n = 37). B. The CAR transgenic (n = 17) vs. wild-type eyes (n = 73).

To investigate the role of the rod cells in generating circadian melatonin rhythmicity, we dissected eyes of the XOP-XCLΔQ-GFP tadpoles and performed eyecup perfusion culture for 5 days [23]. Timed fractions of culture media were collected and melatonin levels were measured by RIA, as previously described [15]. While eyecups from most of the wild-type tadpoles demonstrated normal circadian rhythms of melatonin secretion (Fig. 2A; [15], the majority of the eyecups from the XOP-XCLΔQ-GFP tadpoles show arrhythmicity, demonstrating that disruption of CLOCK selectively in the rod cells alters rhythmicity (Fig. 2B, Table 1). Although melatonin rhythms were altered/abolished in 57.6% of the transgenic eyes, the average melatonin levels (averaged over all time points) were not significantly altered compared to the wild-type eyecups (Fig. 3A). This is consistent with our previous observation using IRBP-XCLΔQ-GFP transgenic eyes in which XCLΔQ is expressed in both rods and cones [15], suggesting that XCLΔQ disrupts circadian rhythmicity without affecting average levels of melatonin synthesis. It is of note that the majority of the XOP-XCLΔQ-GFP tadpoles showed arrhythmicity in circadian melatonin rhythms. The frequency of arrhythmia in these transgenic eyes is lower than that observed in the IRBP-XCLΔQ-GFP eyes where 71% showed abnormal rhythms (arrhythmia or longer period) [15], suggesting that disruption of the clock only in rods is less severe.

**Table 1:** Summary of the circadian melatonin rhythmicity in the two different genotypes.

Genotype	*N*	Period +/− SEM	% Arrhythmic
XOP-XCLΔQ	33	24.1+/−0.6	57.6%***
Wild type	43	24.4+/−0.2	11.6%
CAR-XCLΔQ	31	24.2+/−0.3	29.0%
Wild type	107	24.1+/−0.1	15.0%

***P<0.001.

### Cone-specific XCLΔQ expression does not significantly alter melatonin rhythms

**Figure 4:**
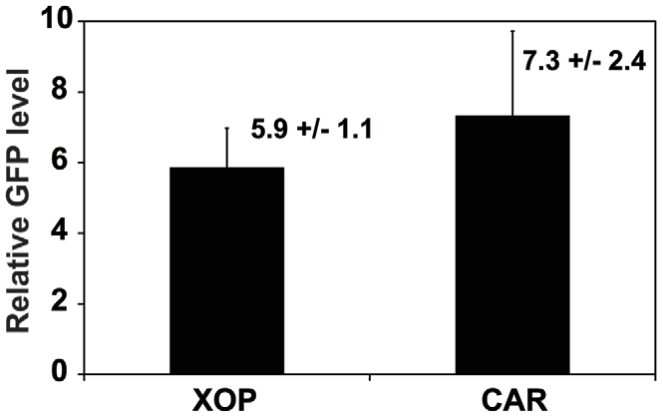
Expression levels of the two different transgenes are comparable. After flow-through culture was complete, each pair of eyes was collected, RNA was extracted and real-time PCR was performed on GFP to compare relative levels of transgene expression. The average GFP levels from the XOP (n = 25) and CAR (n = 26) transgenic eyes were comparable and the difference was not statistically significant. Values are average relative GFP expression levels +/− SEM.

In *Xenopus laevis*, cone cells constitute nearly 50% of the photoreceptor layer cells, a percentage much higher than in human retinas (only about 3% cone cells). Expression of the known clock genes is observed both in rods and cones in the *Xenopus* retinal photoreceptors [17]. These lines of evidence raise the possibility that not only rods but also cones contribute to circadian rhythm generation (e.g., melatonin release). To study the involvement of the circadian clocks in cone cells in generation of circadian rhythms in melatonin release, we generated CAR-XCLΔQ-GFP tadpoles and performed eyecup perfusion culture as described above. The percent of arrhythmic retinas in this group was significantly lower than observed in the XOP- XCLΔQ-GFP retinas (29% vs. 59%; Table 1) and were not significantly different than WT retinas (*P*>0.05, Table 1). The overall melatonin levels (averaged over all time points) in the transgenic eyecups were not significantly different from those in the eyecups from the wild-type siblings (Fig. 3B) or XOP-XCLΔQ-GFP eyecups (Fig. 3B, compare to 3A). To determine whether the difference in melatonin rhythmicity between the XOP-XCLΔQ-GFP and CAR-XCLΔQ-GFP eyes could be due to different expression levels of the transgene, we compared the average expression levels of the transgene (XCLΔQ-GFP) between XOP-XCLΔQ-GFP and CAR-XCLΔQ-GFP transgenic eyecups by quantitative PCR (qPCR). RNAs from the transgenic eyecups used for flow-through culture were extracted and real-time PCR was performed using GFP primers. Although there was variation between individual animals, there was no significant difference in the average XCLΔQ-GFP mRNA levels between the two genotypes, suggesting that the increased arrhythmia in the XOP-XCLΔQ-GFP retinas was not due to higher expression of the transgene (Fig.4).

### Arrhythmia correlates with expression levels of XCLΔQ-GFP in the XOP but not CAR transgenic eyecups

**Figure 5:**
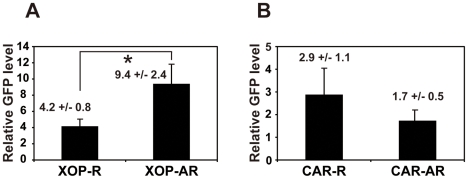
Arrhythmic melatonin secretion correlates with mRNA levels of XOP-XCLΔQ, but not CAR-XCLΔQ. qPCR was performed on GFP as described in Figure 4. The GFP mRNA levels from the arrhythmic and rhythmic animal groups in each of the two transgenic animals were averaged. A. Comparison of GFP levels between rhythmic (XOP-R; n = 13) and arrhythmic (XOP-AR; n = 12) groups in the XOP transgenics (*P<0.05*, Student *t-*test). B. Rhythmic (CAR-R; n = 8) and arrhythmic (CAR-AR; n = 11) groups in CAR transgenic eyecups expressed comparable levels of GFP. Values are average relative GFP expression levels +/− SEM.

Our results from the two different transgenic *Xenopus* as mentioned above raised the question of whether variability of the phenotypes in individual animals in each genotype (i.e., arrhythmic vs. rhythmic melatonin release; Table 1) is due to differential levels of transgene expression. To address this, we performed qPCR using GFP primers as described above (Fig. 4) and compared XCLΔQ-GFP mRNA levels of rhythmic and arrhythmic groups in the same genotype (XOP or CAR). Fig. 5 shows average GFP mRNA level of each transgenic group (rhythmic or arrhythmic animals). Although individual animals in both genotypes exhibited variable expression levels of GFP, in the XOP-XCLΔQ-GFP transgenics, average GFP expression level of arrhythmic group was significantly higher than that of rhythmic group (Fig. 5A; *P<0.05*). In contrast, there was no statistical difference in the GFP mRNA levels between rhythmic and arrhythmic CAR-XCLΔQ-GFP transgenic animals (Fig. 5B). These data indicated that expression level of XCLΔQ in the rod photoreceptors and melatonin arrhythmia were positively correlated, whereas this was not the case for the cone photoreceptors.

## Discussion

In this study, we generated two different transgenic *Xenopus* tadpoles driving XCLΔQ expression in a cell type-specific manner, targeting expression to the retinal rod photoreceptor cells (XOP-XCLΔQ-GFP) or cone photoreceptor cells (CAR-XCLΔQ-GFP). The percentage of arrhythmia in the retinal melatonin release was significantly higher in XOP (57.6%) compare to CAR (29%) or wild-type controls (11%; Table 1). As compared to our previous data where overexpression of XOP-XCLΔQ in both rods and cones resulted in 71.0% of arrhythmicity of melatonin secretion [15], rod-specific (XOP) or cone-specific (CAR) XCLΔQ expression in this study resulted in a significantly lower percentage of arrhythmia (Table 1). Taken together, our studies suggest that both rod and cone photoreceptors contribute to the regulation of circadian rhythmicity of melatonin release in the retina, and that contribution of the rod photoreceptors to the rhythmicity is substantial, whereas that of cone cells is subordinate.

Although the temporal pattern of melatonin release was disrupted to varying degrees in the two different transgenic retinas, average total melatonin levels of both transgenics were comparable to those of wild-types (Fig. 3). These data suggest that the effect of XCLΔQ on melatonin rhythms is not due to its toxicity or its influence on cell metabolism in the photoreceptor cells, and is consistent with our previous observation in which disruption of CLOCK in all the photoreceptors does not affect melatonin production levels [15]. Overall, our present data confirm our previous results that the circadian clock(s) in the retinal photoreceptors governs circadian melatonin rhythms without affecting absolute levels of melatonin synthesis.

Although the majority of the rod-specific (XOP) transgenic eyes showed arrhythmic melatonin secretion, about 40% of the transgenic eyes still retained circadian rhythmicity. The likely explanation for the different effects of CLOCK (XCLΔQ) in individual animals is that the effectiveness of the ablation of the clock function is highly sensitive to the levels of the XCLΔQ expression. We have previously shown *in vitro* that the ability of XCLΔQ to repress endogenous CLOCK-mediated transactivation was dose-dependent and that the level of XCLΔQ expression in rods and cones strongly correlates with the loss of melatonin rhythmicity [15]. Consistent with our previous data, the present study demonstrates that there is a significant correlation between the XCLΔQ expression level and arrhythmicity of melatonin secretion in the rod photoreceptors (Fig. 5A). In contrast, however, the correlation was not observed in the cone-specific XCLΔQ expression (CAR- XCLΔQ transgenics; Fig. 5B), in which there was no statistically significant difference in the XCLΔQ levels between arrhythmic and rhythmic eyecups. Although the reason responsible for this discrepancy remains to be elucidated, the result, along with our present data showing that rod-specific ablation of CLOCK (XCLΔQ overexpression) has a higher effect on melatonin arrhythmia (Table 1), suggests that the circadian clock in the rod photoreceptors plays a predominant role in regulating circadian melatonin rhythms.

Our study raises the question of whether those two photoreceptor cells act independently on the circadian regulation of melatonin secretion, or whether they interact and cooperate with each other. *Xenopus* retina is constituted from approximately the same number of rod and cone cells, and both rods and cones express the melatonin synthetic enzyme AA-NAT at comparable levels [16]–[19]. If rods and cones independently regulate the circadian clock driving melatonin rhythms, and disruption of the clock in either cell-type does not affect circadian rhythms of the other, then one would expect a damping of the melatonin rhythms resulting from half of the cells maintaining rhythmicity while the other half became arrhythmic. Our results are inconsistent with this assumption, and rather raise the possibility that the clocks in the two different cell-types interact with each other. Regarding this possibility, previous reports have demonstrated that the two cell-types are unlikely to be associated by direct neuronal connection, but instead by gap junctions [24]–[27]. Another report in fish and mammals suggested that the circadian clock in the retina regulates gap junction-mediated rod-cone coupling by activating dopamine D_2_-like receptors during the day, so that rod-cone coupling is weak during the day but strong at night [28]. Based on these observations, our data imply that rod and cone photoreceptors interact with each other via gap junctions to coordinate circadian clocks in the individual photoreceptors, and orchestrate circadian physiology such as melatonin release.

Another question raised from our study would be how rod cells dominantly affect circadian rhythms of melatonin release in the retinal photoreceptor layer. Since rods and cones function under different lighting conditions (dim light versus bright light), it is feasible that the retinal circadian clock controlling melatonin rhythmicity responds to light through different cells in a time-of-day-dependent manner. Considering the fact that melatonin is secreted only at night and suppressed by light and dopamine during the day, it is reasonable to suggest that the “rod clock” plays a major role in increasing melatonin release at night. Alternatively, it is also conceivable that the clock in the rods, which are more sensitive to light as compared with the cone clock and can respond to even dim light at night, dominates in the regulation of the retinal circadian physiology. Interestingly, a recent study demonstrated in mammals that the retinal circadian clock regulates the conductance of rod-cone coupling via gap junctions, so that strong coupling increases signal flow from rods to cones during the night, but not during the day [29]. These data suggested that this circadian clock-controlled neural pathway from rods to cones, followed by that from cones to horizontal cells, results in the responses of not only rods but also cones to dim light at night. Taken together, our data along with previous studies raises a possibility that the circadian regulation of melatonin release by the rod clock is dominant, but the cone clock, which is strongly coupled to rods and perceives rod input, is also involved in the regulation. Further studies need to be done to elucidate how the two photoreceptor clocks interact and control circadian outputs including melatonin rhythm.

Consistent with the observations in *Xenopus,* mammalian retinas also exhibit circadian melatonin rhythms [30]. On the other hand, recent studies in mammals suggest that circadian clock(s) regulating several aspects of retinal physiology is located in the inner retina, which is totally different from that in *Xenopus.* [31]–[33]. In contrast to *Xenopus* retina where photoreceptors predominantly express clock/clock-related genes, mammalian retinas primarily express core clock genes in other cell types such as dopaminergic amacrine cells, horizontal cells, and ganglion cells [33]. These data raise a possibility that, while the roles of ocular clock in vertebrates are well conserved during evolutional processes, both localization of circadian clock(s) and interactions between clock cells to regulate retinal circadian physiology are different. Based on these studies, it is possible that the basic circadian organizations/systems of the retina in *Xenopus* and in mammals are fundamentally uncommon. On the other hand, commonality between the mammalian and amphibian retinal circadian organizations cannot be excluded, since a recent report suggested that mammalian photoreceptors contain the circadian pacemaker driving rhythmic melatonin synthesis [34]. To address the question of whether the circadian system in *Xenopus* as described in this study can be applied to the mammals, it would be necessary to analyze the mammalian ocular system on parallel approaches.

In summary, we have successfully generated transgenic *Xenopus* targeting dominant negative CLOCK expression specifically to rod or cone photoreceptor cells. Only recently, significant roles of peripheral circadian clock have been reported by tissue-specific genetic manipulation of circadian clock [4]–[5], [35]–[36]. However, targeted genetic engineering to restricted cell-type is still difficult in many tissues/organs because of lack of appropriate cell type-specific promoters. This technique will pave the way for a comprehensive understanding of the organization of the retinal circadian clock in all vertebrate species. Moreover, not only in the retina but also in any tissues/organs, this fine molecular dissection of the specific cell type as we report here will be a useful tool to provide detailed information such as identification of a particular cell-type(s) involved in certain physiology, understanding precise interactions or functional assignment among different cell-types.

## Methods

### DNA constructs

The constructs, XOP-XCLΔQ-GFP and CAR-XCLΔQ-GFP, were made as previously described [15], but using the XOP and CAR promoters, respectively [16], [21]

### Generation of transgenic tadpoles

*Xenopus laevis* adults were purchased from NASCO (Fort Atkinson, WI) to obtain eggs and sperm for transgenesis. Restriction enzyme-mediated integration (REMI) methods, in which the transgene is stably inserted into the sperm genome followed by fertilization of eggs with the sperm nuclei, was modified and used for making transgenic tadpoles as described previously [15], [22]. Developing embryos were maintained in 12 hr light/12 hr dark (LD 12∶12) cycles until they reached the appropriate age for analysis (2–3 weeks).

### Genotyping

Genomic DNA was extracted from the tail tip from each tadpole using DNeasy Tissue Kit (Qiagen, Valencia, CA), and PCR was performed using GFP-specific primers: 5′-CAAGCTGACCCTGAAGTTCATCTG-3′ and 5′-CGGATCTTGAAGTTCACCTTGATG-3′. PCR conditions were as follows: 95°C for 10 min, 30 cycles of 94°C for 40 sec, 55°C for 1 min, 72°C for 1 min; and 72°C for 10 min.

### Perfusion culture of eyecups

Eyes from 2- to 3-week old tadpoles (entrained in LD 12: 12 cycles) were dissected before dark onset, and the cornea and lens were removed. Eyecups were then cultured in a perfusion chamber in constant darkness (DD) as previously described. The culture plates were kept in light–tight chambers, and the medium was continuously delivered with a syringe pump (model 2000, Harvard Apparatus, Holliston, MA) to each well at a constant rate of 0.2 ml/hr. Superfusates were collected in a fraction collector over 4 hour intervals.

### Melatonin Measurement

Radioimmunoassay (RIA) was performed to determine melatonin levels in the superfusate samples from flow-through culture as previously described [37] and validated for measurement of melatonin (ruling out cross-reactivity to other related compounds) in our culture medium [23].

### Period Analysis

Circadian rhythmicity of melatonin release was evaluated using a fast Fourier transform-nonlinear least squares (FFT-NLLS) estimation method as previously described [15]. We classified eyecups as rhythmic if the relative amplitude of the period was less than 1 (FFT-NLLS default setting).

### Quantitative PCR (qPCR)

After flow-through culture was complete, culture medium was removed, eyecups were homogenized, and total RNA was isolated using Trizol reagent (Life Technologies, Gaithersburg, MD) following the manufacturer's instructions. RNA was then reverse-transcribed into cDNA using Superscript II (Life Technologies, Gaithersburg, MD) and used as a template for PCR. Real-time quantitative PCR was performed using GeneAmp 7700 Sequence Detection System and SYBR Green Master Mix that includes SYBR Green Dye and AmpliTaq Gold (PE Applied Biosystems, Foster City, CA) according to manufacturer's instructions. For the quantitation of the transgene expression, GFP-specific primers were used for the PCR reaction (5′-AGCAAAGACCCCAACGAGAA-3′, 5′-GGCGGCGGTCACGAA-3′). Human 18S rRNA primers (PE Applied Biosystems, Foster City, CA; 5′-CGGCTACCACATCCAAGGAA-3′, 5′-GCTGGAATTACCGCGGCT-3′) were used as an endogenous control for purpose of normalization. For each experiment, a standard curve was prepared for each primer set using as template dilution series of cDNA from transgenic eyes, where the most concentrated standard was assigned an arbitrary value of 10. The levels of GFP and 18S rRNA levels in each test sample were then determined based on the standard curve. We then normalized the GFP expression levels to the 18S rRNA expression levels for each pair of eyes. The data shown are the averages of three or six independent measurements for each tadpole.
